# Opportunistic screening for alcohol use problems in adolescents attending emergency departments: an evaluation of screening tools

**DOI:** 10.1093/pubmed/fdy049

**Published:** 2018-03-26

**Authors:** Simon Coulton, M Fasihul Alam, Sadie Boniface, Paolo Deluca, Kim Donoghue, Eilish Gilvarry, Eileen Kaner, Ellen Lynch, Ian Maconochie, Paul McArdle, Ruth McGovern, Dorothy Newbury-Birch, Robert Patton, Ceri J Phillips, Thomas Phillips, Hannah Rose, Ian Russell, John Strang, Colin Drummond

**Affiliations:** 1 Centre for Health Services Studies, School of Social Science and Social Policy Research, George Allen Wing, University of Kent, Canterbury, Kent, UK; 2 College of Health Sciences, Qatar University, Doha, Qatar; 3 Addictions Department, National Addiction Centre, Institute of Psychiatry, Psychology and Neuroscience, King’s College London, London, UK; 4 Northumberland Tyne and Wear NHS Foundation Trust, Newcastle, UK; 5 Institute of Health and Society, Newcastle University, Newcastle, UK; 6 Paediatric Emergency Medicine, Imperial College London, London, UK; 7 School of Health and Social Care, Teesside University, Middlesbrough, UK; 8 School of Psychology, University of Surrey, Guildford, UK; 9 Swansea Centre for Health Economics, College of Human and Health Sciences, Swansea University, Swansea, UK; 10 University of Hull, Hull, UK; 11 Swansea University Medical School, Swansea, UK

**Keywords:** adolescent, alcohol, diagnosis, screening

## Abstract

**Objective:**

To estimate and compare the optimal cut-off score of Alcohol Use Disorders Identification Test (AUDIT) and AUDIT-C in identifying at-risk alcohol consumption, heavy episodic alcohol use, ICD-10 alcohol abuse and alcohol dependence in adolescents attending ED in England.

**Design:**

Opportunistic cross-sectional survey.

**Setting:**

10 emergency departments across England.

**Participants:**

Adolescents (*n* = 5377) aged between their 10th and 18th birthday who attended emergency departments between December 2012 and May 2013.

**Measures:**

Scores on the AUDIT and AUDIT-C. At-risk alcohol consumption and monthly episodic alcohol consumption in the past 3 months were derived using the time-line follow back method. Alcohol abuse and alcohol dependence was assessed in accordance with ICD-10 criteria using the MINI-KID.

**Findings:**

AUDIT-C with a score of 3 was more effective for at-risk alcohol use (AUC 0.81; sensitivity 87%, specificity 97%), heavy episodic use (0.84; 76%, 98%) and alcohol abuse (0.98; 91%, 90%). AUDIT with a score of 7 was more effective in identifying alcohol dependence (0.92; 96%, 94%).

**Conclusions:**

The 3-item AUDIT-C is more effective than AUDIT in screening adolescents for at-risk alcohol use, heavy episodic alcohol use and alcohol abuse. AUDIT is more effective than AUDIT-C for the identification of alcohol dependence.

## Introduction

The excessive consumption of alcohol is a major global public health issue^1,2^ and places a significant burden on international health systems. While the majority of this burden lies with adult populations, for many the roots of problematic alcohol use lies in adolescence.^3^ Adolescence is a critical developmental stage when young people make behavioural and lifestyle choices that have the potential to impact on their health and wellbeing into adulthood. Inappropriate risk-taking is significantly associated with health and social harm during adolescence.^4^ Young people are much more vulnerable than adults to the adverse effects of alcohol use due to a range of physical and psychological factors that often interact. Adolescence is also a unique period whereby neural proliferation and subsequent ‘pruning’ processes may leave brain structures particularly vulnerable to the effects of alcohol.^5,6^

A recent survey of alcohol consumed by 14–15 years old across 36 European countries reported that in the United Kingdom (UK) 87% had consumed alcohol at least once in their lifetime and 57% had consumed alcohol at least once in the past month.^7^ The prevalence of consuming alcohol increases with age, with data from 2016 indicating that 9% of boys aged 11–15 years, and 11% of girls had consumed alcohol in the past 7 days. Of these, 1% of 11 years old consumed alcohol in the past 7 days, increasing to 24% at age 15. In terms of quantity of alcohol consumed in the past 7 days mean consumption was 10.3 units for boys and 8.9 units for girls aged 11–15 years.^8^

An evidence based review of the risks and harms of alcohol consumption in young people^9^ provided a basis for the Chief Medical Officer for England recommendations for alcohol consumption in young people—that young people up to the age of 15 abstain completely from drinking and those aged 15–17 are advised not to drink, but if they do drink, they should not exceed 2–3 standard drinks in any day and no more than once per week.^10^

While there is a body of evidence addressing the effects of school based interventions for delaying the onset of drinking in adolescents,^11^ and some evidence for interventions to delay the age of onset or reduce alcohol consumption for adolescents in other settings,^12,13^ there exists a paucity of evidence of the effectiveness of interventions to reduce adolescent alcohol use in primary care settings. Recommendations from the World Health Organization, US Surgeon General and American Academy of Paediatrics advocate that more evidence is needed on the effectiveness of opportunistic screening and interventions for adolescents who consume alcohol^14,15^ and this population has been identified as a key target group for the reduction of alcohol use and related harm^16,17^ in both English and Scottish alcohol strategies.

The identification of adolescents who consume alcohol at problematic levels is a key element in any screening and intervention strategy. To offer such interventions practitioners need access to screening tools that are high in both sensitivity and specificity and are quick and easy to apply at minimal cost. Biochemical markers of alcohol use such as ϒ-glutamyltransferase, aspartate aminotransferase, erythrocyte mean cell volume and percent carbohydrate deficit transferrin are impractical and of little use in this population and have been found to be inferior to short paper instruments in adult populations.^18^ The Alcohol Use Disorders Identification Test (AUDIT)^19^ is a 10-item self-completion instrument with established diagnostic properties for problematic alcohol use in adults that addresses three domains of alcohol-related problems; consumption, negative consequences and symptoms of dependence. AUDIT is one of the few screening instruments that specifically incorporates consumption into the scoring algorithm and may be particularly suitable for adolescents who are more likely to experience a range of alcohol-related problems as a result of consumption rather than psychological consequences of alcohol use. Further, it may be the case that the three specific alcohol consumption questions, AUDIT-C, may be equally efficient as a brief screening instrument as the full AUDIT. Previous studies suggest that the AUDIT may be more useful than other brief screening instruments in adolescent populations, but there is less consensus regarding appropriate cut-off points for different severities of alcohol use^20–25^ and no previous research has compared the relative effectiveness of AUDIT versus AUDIT-C as opportunistic screening approaches for adolescent populations. Much of the prior research has aimed to compare the performance of a variety of different screening instruments^21,26–28^ against more severe clinical alcohol use disorder criteria whereas adolescents are more likely to experience alcohol-related difficulties at lower levels of consumption and this is in part due to the pattern of consumption in the form of heavy episodic alcohol use.^29^ In addition, the majority of studies have been conducted in older adolescent populations^20,22^ and often involve college students, primary care or hospitalized participants, rather than an opportunistic sample and are limited in their generalizability to the wider adolescent population and particularly limited in their generalizability to the UK.

Our aim was to estimate and compare the sensitivity, specificity, and diagnostic odd ratio of the AUDIT and AUDIT-C in identifying at-risk alcohol use, monthly heavy episodic alcohol use, alcohol abuse and alcohol dependence in the context of an opportunistic screening programme for adolescents, aged between 10 and 17 years, attending emergency departments (ED) in England. To be acceptable as a screening test in clinical practice we expected the sensitivity and specificity at a selected cut-point would exceed 0.70.

## Methods

The study was conducted in accordance with ethical approval from the National Health Service Multi-Centre Research Ethics Committee (ref: 12/L0/0799) and was registered in an appropriate trial registry (ref: ISRCTN 45300218).

### Design

An opportunistic cross-sectional survey conducted between December 2012 and May 2013 across 10 ED’s in England, encompassing a mix of metropolitan urban and rural centres across the North East, Yorkshire and Humber, London and the South. Consecutive attendees, between the hours of 8 am and midnight were approached by trained researchers after the initial triage assessment.

Researcher assessment was conducted blind to the results of the screening measure and the order of presentation of all measures was randomized using random permuted blocks of random length and embodied within the electronic data collection tool, stratified by age and centre. All assessment instruments used a 3-month assessment time-frame.

### Measures

#### Gold standard measures

To elicit the gold-standard measures of at-risk drinking and monthly heavy episodic alcohol use we used the Time-Line Follow Back −90 days (TLFB90). This is a reliable and valid method to ascertain the frequency and quantity of alcohol consumed in clinical and non-clinical populations for periods ranging from 1 to 365 days.^30^ The method has established psychometric properties for adolescent populations^31^ and is conducted by a trained researcher and the 90-day version takes ~30 min to complete. The responses to the interview are converted to UK standard drinks and can be used as either continuous or categorical outcomes. At-risk drinking was defined as consuming three or more standard drinks, where a standard drink equates to 8 g of pure ethanol, in a single day in the past 90 days. Monthly heavy episodic alcohol consumption was defined as consuming six or more standard drinks in a single drinking episode in each month over the past 3 months.

MINI-KID has established validity and reliability in the identification of psychiatric diagnoses for children and adolescents.^32^ The alcohol use module consists of seven detailed questions that diagnose both alcohol abuse and alcohol dependence in accordance with ICD-10 criteria.

### Screening tools

The AUDIT^19^ is a 10-item self-completion questionnaire that measures the quantity and frequency of alcohol consumption, drinking behaviour, alcohol-related problems and the symptoms of alcohol dependence. Each item is scored 0–4 and summed to create an overall score with a maximum of 40. The instrument is widely used in adult populations and a cut-off score of 8 or more has high levels of sensitivity (92%) and specificity (94%) for at-risk drinking in adult populations.^19^ The AUDIT-C^33^ consists of the three consumption items of AUDIT and has been validated as a short-screen in adults, AUDIT-C scores range from 0 to 12, with five or more being indicative of at-risk alcohol use.

### Participant recruitment

To be included in the survey, participants had to be aged between their 10th and 18th birthday, alert and orientated and able to communicate in English sufficiently to complete the survey. Participants were excluded if they had a severe injury requiring immediate intervention, were grossly intoxicated, had a serious mental health presentation or if they, or their parent or guardian, refused to provide consent.

Participants were provided with the study information sheet and allowed to ask any questions prior to providing consent. Where a child was aged 16 years or less Gillick competency was assessed^34^ by a member of the clinical staff in the ED, and where a participant was not found competent consent was sought from the parent or carer. If a parent or carer was present with the child, parent consent was sought in addition to child consent. The survey was conducted in a private area of the ED with a trained researcher who was available to answer any questions and provide appropriate assistance. The survey was anonymous and self-completed using an electronic tablet device with the exception of the time-line follow back interview (TLFB)^30^ that was conducted by the researcher. At the end of the survey participants were thanked for their time and returned to the care of the ED, were provided with an age-appropriate alcohol awareness leaflet and given a £5 gift voucher for participating.

### Statistical methods

We compiled and analysed the results using STATA14. The influence of potential covariates of age and gender, and clustering by ED, were incorporated into the analysis using the ROCREG function. We constructed receiver operator characteristic curves on the basis of all continuous values of the test results for AUDIT and AUDIT-C compared with each of the gold-standards; at-risk drinking, monthly heavy episodic alcohol use, alcohol abuse and alcohol dependence. We estimated the sensitivity and specificity of each cut-off point and generated the diagnostic odds ratio and associated 95% confidence interval. The diagnostic odds ratio was used to estimate optimal cut-points and is a measure of effectiveness of a dichotomous classification that is the ratio of the odds of being positive if truly positive relative to the odds of being positive if truly negative. It has advantages over other methods of diagnostic test effectiveness in that it is less susceptible to statistical artefacts, a criticism of the Youden Index, and does not rely on the sample prevalence, making it more useful for comparison across different study samples.^35^

## Results

Overall 5781 participants were asked to participate in the survey of whom 5377 (93%) consented to participate across the 10 ED’s. The mean age was 13.3 (SD 2.1) years with similar proportions of male (53.7%) and female (46.3%) participants and the majority White (72.6%). Overall 2112 (39.3%) had consumed alcohol at some time in the past and 1378 (25.6%) had consumed alcohol in the past 3 months. Those who had consumed alcohol tended to be older (14.8 versus 12.3 years) and were more likely to be white (83.4 versus 65.6%) (Table 1).

**Table 1:** Demographic variables in 5377 adolescent attendees overall and by drinking status

Variable	All attendees (*n* = 5377)	Drinkers (*n* = 2112)	Non-drinkers (*n* = 3265)
Mean age (SD)	13.28 (2.07)	14.77 (1.64)	12.33 (1.74)
Age 10, *n* (%)	570 (10.6)	24 (1.1)	543 (16.8)
Age 11, *n* (%)	701 (13.0)	50 (2.4)	647 (20.0)
Age 12, *n* (%)	809 (15.0)	133 (6.3)	668 (20.6)
Age 13, *n* (%)	845 (15.7)	248 (11.7)	595 (18.4)
Age 14, *n* (%)	751 (14.0)	387 (18.3)	363 (11.2)
Age 15, *n* (%)	784 (14.6)	502 (23.8)	276 (8.5)
Age 16, *n* (%)	534 (9.9)	428 (20.3)	105 (3.2)
Age 17, *n* (%)	382 (7.1)	340 (16.1)	40 (1.2)
Male, *n* (%)	2886 (53.7)	1093 (51.8)	1793 (54.9)
Ethnicity, *n* (%)			
White	3726 (72.6)	1687 (83.4)	2039 (65.6)
Black	698 (13.6)	150 (7.4)	548 (17.6)
Chinese	4 (0.1)	1	3 (0.1)
Mixed	289 (5.6)	97 (4.8)	192 (6.2)
Asian	255 (5.0)	35 (1.7)	220 (7.1)
Other	144 (2.8)	45 (2.2)	99 (3.2)
Mode of arrival, *n* (%)			
Own means	3953 (74.0)	1667 (79.1)	2286 (70.6)
Ambulance	331 (6.2)	143 (6.8)	188 (5.8)
Police	2 (0.05)	2 (0.1)	0
Other	1059 (19.8)	295 (14.0)	764 (23.6)
Smoker, *n* (%)	481 (9.0)	455 (21.6)	26 (0.8)
Consumed alcohol in the past 3 months, *n* (%)	1378 (25.6)	1378 (64.9)	0

Using the sample to estimate the prevalence of drinking behaviours in adolescents attending ED, the prevalence of at-risk drinking was 14.8% (95% CI: 13.9–15.8%). The prevalence of monthly heavy episodic alcohol use was 10.6% (9.8–11.4%), alcohol abuse 2.4% (2.0–2.8%) and alcohol dependence 1.2% (0.9–1.5%). In the sample of those who had consumed alcohol in the past 3 months the prevalence of these behaviours was significantly higher (Table 2).

**Table 2:** Alcohol-related variables for all participants and those who consumed alcohol in the past 3 months

Variable	All participants (*n* = 5377)	Those who consumed alcohol in past 3 months (*n* = 1378)
Consumed alcohol in past 24 h, *n* (%)	115 (2.1)	115 (8.5)
Mean age in years (SD)	13.28 (2.07)	15.12 (1.51)
Mean age of first drink in years (SD)	12.74 (2.24)	12.90 (2.17)
Total alcohol consumed in past 3 months in standard units^a^ (SD)	7.19 (39.47)	33.09 (79.28)
Hazardous alcohol consumption in past 3 months^b^, *n* (%)	796 (14.8)	796 (67.9)
Heavy episodic alcohol consumption in past 3 months^c^, *n* (%)	572 (10.6)	572 (48.8)
Alcohol abuse^d^, *n* (%)	127 (2.4)	127 (9.2)
Alcohol dependent^d^, *n* (%)	67 (1.2)	67 (5.0)
Mean AUDIT score (SD) (values can range from 0 to 40 with higher scores indicative of greater problems)	1.18 (1.78)	4.83 (5.03)
Mean AUDIT-C score (SD) (values can range from 0 to 12 with higher scores indicative of greater problems)	0.75 (3.23)	2.98 (2.46)

^a^Standard unit equivalent to 8 g of ethanol.

^b^Hazardous consumption defined as drinking three or more standard units in a single day.

^c^Heavy episodic consumption defined as drinking six or more standard units in a single drinking episode.

^d^Using ICD-10 criteria using MINI-KID.

A significant positive correlation was identified for AUDIT score with the total number of standard drinks consumed in the past 3 months (Spearman rho, *r* = 0.72, 95% CI: 0.71–0.73; *P* < 0.001) and a similar correlation identified for AUDIT-C score (*r* = 0.69, 95% CI: 0.68–0.70; *P* < 0.001). Screening properties of the questionnaire were tested against the gold standard criteria for at-risk drinking, heavy episodic alcohol consumption, alcohol abuse and alcohol dependence. Screening results for all cut-points were assessed and the results of those around the optimal cut-point are reported in Table 3.

**Table 3:** Area under the receiver operator curve (AUC), sensitivity, specificity and diagnostic odd ratio of AUDIT and AUDIT-C cut-points for hazardous drinking, monthly episodic alcohol use, alcohol abuse and alcohol dependence for 5377 adolescent attendees at ED

Outcome	Prevalence %	AUC	Sensitivity %	Specificity %	Diagnostic odd ratio
	(95% CI)	(95% CI)	(95% CI)	(95% CI)	(95% CI)
At-risk/hazardous drinking	15 (14; 16)				
AUDIT					
≥3		0.81 (0.79; 0.94)	78 (75; 82)	94 (94; 95)	55 (47; 87)
≥4		0.81 (0.79; 0.94)	75 (72; 78)	98 (98; 99)	147 (126; 351)
≥5		0.84 (0.82; 0.87)	65 (61; 69)	98 (98; 99)	91 (77; 220)
AUDIT-C					
≥2		0.84 (0.82; 0.87)	91 (88; 93)	89 (87; 91)	81 (49; 134)
≥3		0.98 (0.97; 0.99)	89 (86; 91)	97 (96; 97)	261 (147; 242)
≥4		0.98 (0.97; 0.98)	72 (68; 77)	97 (96; 97)	83 (51; 108)
Monthly episodic use	10 (10; 11)				
AUDIT					
≥3		0.92 (0.90; 0.95)	80 (77; 82)	92 (89; 95)	46 (27; 86)
≥4		0.87 (0.84; 0.91)	78 (74; 81)	97 (97; 98)	114 (92; 109)
≥5			58 (54; 63)	98 (94; 99)	67 (18; 168)
AUDIT-C					
≥2			82 (79; 85)	89 (87; 90)	37 (25; 51)
≥3			76 (73; 80)	98 (97; 98)	155 (87; 196)
≥4			61 (57; 66)	99 (96; 99)	77 (32; 192)
Alcohol abuse	2 (2; 3)				
AUDIT					
≥3			94 (88; 97)	85 (82; 88)	88 (33; 237)
≥4			93 (87; 96)	88 (87; 89)	97 (44; 194)
≥5			83 (75; 88)	92 (91; 93)	56 (30; 97)
AUDIT-C					
≥2			91 (85; 95)	85 (84; 86)	57 (30; 116)
≥3			91 (85; 95)	90 (88; 91)	91 (42; 192)
≥4			65 (56; 73)	93 (92; 93)	25 (15; 36)
Alcohol dependent	1 (1;2)				
AUDIT					
≥6			96 (89; 99)	92 (90; 94)	276 (73; 1551)
≥7			96 (89; 99)	94 (95; 95)	376 (154; 1881)
≥8			91 (81; 96)	95 (95; 96)	192 (81; 576)
AUDIT-C					
≥4			85 (79; 88)	92 (91; 93)	65 (43; 97)
≥5			80 (67; 89)	95 (95; 95)	76 (39; 154)
≥6			67 (55; 77)	97 (96; 97)	65 (39; 108)

The optimum cut-off point for AUDIT in identifying either at-risk drinking, monthly heavy episodic drinking or alcohol abuse was 4 or more, which provided the optimal cut-point to provide acceptable sensitivity, specificity and diagnostic odds. An AUDIT-C score of 3 or more demonstrated almost identical diagnostic properties but with a significantly better sensitivity for at-risk drinking.

An AUDIT score of 7 or more provided a significantly more effective cut-point for alcohol dependence than any other cut-point and demonstrated significantly better diagnostic properties than an AUDIT-C score of 5 or more.

We assessed the potential influence of age, gender and ED on our findings and found these effects to minimal and not statistically significant from our main findings. The results without incorporation of these variables is therefore reported.

## Discussion

### Main findings of this study

A simple short three item self-completed screening instrument, the AUDIT-C, is overall more effective than the longer 10-item AUDIT in identifying adolescents who engage in at-risk of alcohol consumption, monthly heavy episodic alcohol use and fulfil ICD-10 criteria for alcohol abuse. Further the AUDIT with a cut-off score of 7 is more efficient than AUDIT-C in identifying adolescents with alcohol dependence. In addition, AUDIT-C and AUDIT are widely employed as screening tools for adults in clinical and non-clinical settings and these can be applied equally to adolescent populations with these lower cut-off scores. We conclude that AUDIT-C should be employed with this population with a cut-off score of 3 as a positive screen for at-risk drinking, monthly heavy episodic alcohol use and alcohol abuse. For those who score 5 or more on AUDIT-C we recommend the use of the additional 7 questions constituting the full AUDIT be administered. With those scoring 7 or more being clinically assessed for alcohol dependence.

### What is already known on this topic

There is a body of evidence suggesting that interventions for alcohol using adolescents are effective and that they are more effective when targeted as secondary prevention strategies, i.e. at those already engaged in consuming alcohol.^12,13^ A critical first step in the delivery of interventions is employing opportunistic screening tools and the combination of effective screening tools and intervention strategies offers significant potential to reduce the burden of alcohol use on adolescents, health systems and wider society and further consideration should be given to the routine opportunistic implementation of screening strategies for adolescent populations.

### What this study adds

Routine alcohol screening of adolescents should be considered across the UK National Health Service. This study demonstrates that the process can be simplified by using short screening tools already in use for adult populations. This requires appropriate training, resources and incentives for staff. Identifying those adolescents that may benefit from interventions to address alcohol use and associated multiple risk behaviours will help to reduce the burden of alcohol use across the health service and society. This has the potential to enhance the future health of the adolescent population well into adulthood.

### Limitations of this study

Our study was conducted in ED and this could be seen as compromising the generalizability of the findings to other health settings. Yet adolescents are far less frequent attenders at primary care and the ED provides an opportunity to access this population and in turn provides the ‘teachable moment’, that is hypothesized to play a crucial role in effective behaviour change.^36^ Further, we aimed to ensure generalizability of our sample to other ED’s in the UK by including centres covering rural and urban areas and areas with the lowest and highest population prevalence of adolescent alcohol use and areas of high and low socio-economic status. In addition, our estimates of alcohol use problems compare well with national epidemiological surveys, that suggest 27% of adolescents consume alcohol versus 26% in our study, 9% have been drunk three or more times in the past 4 weeks compared with 11% of episodic drinkers in the past 3 months in our study.^37^ We also recognize that those who scored negative on the screening tool and outcome assessments may have misreported their alcohol consumption and we took a variety of steps to ameliorate this by ensuring anonymity and confidentiality. Previous evidence would suggest this form of social desirability bias is limited.^38^ This study was the first study of the screening instruments in a real-life health setting in the UK, one where the burden of alcohol use is a real concern.
